# Molecular and metabolic regulation of immunosuppression in metastatic pancreatic ductal adenocarcinoma

**DOI:** 10.1186/s12943-023-01813-y

**Published:** 2023-07-24

**Authors:** Shailendra K. Gautam, Surinder K. Batra, Maneesh Jain

**Affiliations:** 1 Department of Biochemistry and Molecular Biology, College of Medicine, University of Nebraska Medical Center, Omaha, NE 68198 USA; 2 Fred & Pamela Buffett Cancer Center, University of Nebraska Medical Center, Omaha, NE 68198 USA

**Keywords:** Immunosuppression, Pre-metastatic niche, Exosomes, Non-coding RNA, Microbiome, Immune and metabolic checkpoints

## Abstract

Immunosuppression is a hallmark of pancreatic ductal adenocarcinoma (PDAC), contributing to early metastasis and poor patient survival. Compared to the localized tumors, current standard-of-care therapies have failed to improve the survival of patients with metastatic PDAC, that necessecitates exploration of novel therapeutic approaches. While immunotherapies such as immune checkpoint blockade (ICB) and therapeutic vaccines have emerged as promising treatment modalities in certain cancers, limited responses have been achieved in PDAC. Therefore, specific mechanisms regulating the poor response to immunotherapy must be explored. The immunosuppressive microenvironment driven by oncogenic mutations, tumor secretome, non-coding RNAs, and tumor microbiome persists throughout PDAC progression, allowing neoplastic cells to grow locally and metastasize distantly. The metastatic cells escaping the host immune surveillance are unique in molecular, immunological, and metabolic characteristics. Following chemokine and exosomal guidance, these cells metastasize to the organ-specific pre-metastatic niches (PMNs) constituted by local resident cells, stromal fibroblasts, and suppressive immune cells, such as the metastasis-associated macrophages, neutrophils, and myeloid-derived suppressor cells. The metastatic immune microenvironment differs from primary tumors in stromal and immune cell composition, functionality, and metabolism. Thus far, multiple molecular and metabolic pathways, distinct from primary tumors, have been identified that dampen immune effector functions, confounding the immunotherapy response in metastatic PDAC. This review describes major immunoregulatory pathways that contribute to the metastatic progression and limit immunotherapy outcomes in PDAC. Overall, we highlight the therapeutic vulnerabilities attributable to immunosuppressive factors and discuss whether targeting these molecular and immunological “hot spots” could improve the outcomes of PDAC immunotherapies.

## Introduction

Pancreatic ductal adenocarcinoma (PDAC) is a highly lethal gastrointestinal (GI) cancer characterized by early metastasis, high recurrence, and poor survival [1]. Compared to patients with localized tumors, the 5-year survival rate for metastatic PDAC patients drops significantly from ~ 42% to ~ 3%, which is the worst survival rate in all GI cancers [1]. Resection-based interventional therapies are the only effective treatment modalities to improve survival in PDAC patients, as a recent study reports a 5-year survival rate of 16–18% with single-agent adjuvant chemotherapy and 30–50% with combination adjuvant chemotherapy having gemcitabine (Gem) and capecitabine, Gem and FOLFIRINOX, and Gem plus nab-paclitaxel [2, 3]. Unfortunately, surgical intervention is only possible in 15–20% of PDAC patients who are diagnosed early when the disease is localized or borderline resectable [4, 5]. Nonetheless, most PDAC patients undergoing surgical intervention eventually develop local recurrence or distant metastases. Recently, a retrospective study showed that more than 75% of recurrent PDAC patients developed distant metastases, with or without local recurrence [6], suggesting that the early or recurrent metastatic PDAC predominantly contributes to poor patient survival and poses a significant challenge for the clinical management of PDAC. Previous investigations have identified genetic, epigenetic, and molecular mechanisms that contribute to epithelial to mesenchymal transition of cancer cells, their dissemination from the local site, and the establishment of distant metastases [7–10]. The liver is the most common site of metastasis for PDAC, along with other sites, including the lungs, bone, and brain [11]. Within the pancreatic tumor, only a fraction of cancer cells undergo epithelial to mesenchymal transition (EMT) and develop metastatic traits under various molecular and immunological cues from stromal cells [10]. Immunosuppression is the major stromal and peripheral factor contributing to tumor initiation, progression, metastases, and immunotherapy resistance [12, 13]. Understanding the role of immunosuppression during PDAC progression will be a useful approach to target various mechanisms to alleviate immunosuppression and enhance the efficacy of PDAC immunotherapies.

Immunologically ‘cold’ PDAC is further aggravated by the immunosuppressive tumor microenvironment factors causing the failure of immunotherapy [12, 13]. Previous studies have shown that a low mutational burden and poor immunogenicity correlate with poor response to immunotherapy in PDAC [14, 15]. In addition, tumor intrinsic factors such as extensive fibrosis, a disrupted vasculature, a hypoxic microenvironment, and stromal cytokines not only prevent immune cells from infiltrating into the tumor bed but also lead to immune cell dysfunction creating an immunosuppressive niche [16, 17]. Besides highly immunosuppressive TME, PDAC patients also develop systemic immunosuppression, which provides an opportunity for ‘ready to go’ tumor cells to escape host immune surveillance and disseminate from the localized site and metastasize to distant organs [18]. Thus, it is important to understand the mechanisms and impact of immunosuppression during PDAC initiation, progression, and metastasis for designing effective immunotherapy-focused interventions to target PDAC.

## Immunosuppression during PDAC progression

Regardless of the origins, PDAC represents more than 90% of all diagnosed pancreatic cancer cases [19, 20]. Following oncogenic *KRAS* and subsequent *TP53*, *SMAD4*, and *CDKN2A* mutations, several other factors, such as alcohol abuse, smoking, and chronic inflammation inducing acinar to ductal metaplasia (ADM), have been reported to promote PDAC [20–22]. However, for a transformed cell to survive, it must attain an immunosuppressive phenotype, such as decreased MHCI expression and upregulation of programmed cell death receptor ligand-1 (PD-L1) and CD47, which hinder the anti-tumor immune response by engaging and suppressing the activated T cells and relaying ‘don’t eat me’ signal to the phagocytic macrophages, respectively [23, 24]. In fact, a recent study suggests that constitutively active KRas^G12D^ regulates autophagy-induced MHCI downregulation [25, 26], a major mechanism that PDAC cells employ to escape immune surveillance. This gain of an immunosuppressive phenotype is further aggravated by several immunosuppressive factors that accumulate in the local microenvironments of primary and metastatic PDAC, involving the immunosuppressive stromal cancer-associated fibroblasts (CAFs), immune cells, and the cytokines and chemokines [10, 27]. The tumor immune microenvironment is highly dynamic and constantly evolves during disease progression and metastatic cascade. This change towards an immunosuppressive TME composition is highly context-dependent and regulated by various genetic, epigenetic, metabolic, and immunological factors that determine different PDAC subtypes [16].

**Fig. 1:**
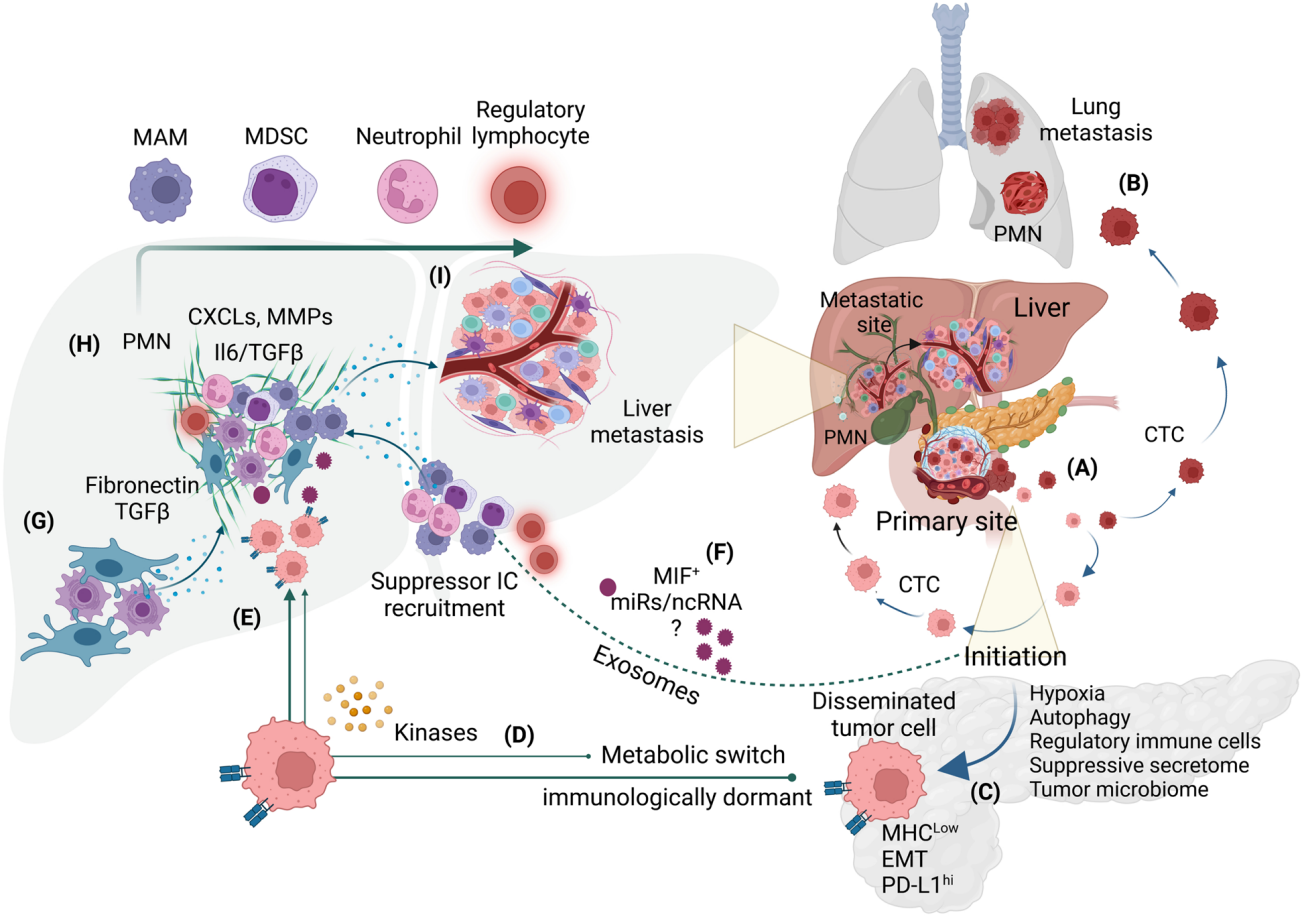
Metastatic cascade during PDAC progression. **A** Cells disseminating from primary tumors preferentially metastasize to different organs such as the liver (**A**) and lung (**B**). **C** Several TME factors such as hypoxia, autophagy, and suppressive cytokines and chemokines influence tumor cells to undergo EMT and gain immunosuppressive phenotype with reduced expression of MHCI and epithelial markers and increased PD-L1 expression. **D**, **E** Immunological dormancy, metabolic switch, and activation of metastasis-associated kinases promote disseminating cells to metastasize to different organs having a pre-metastatic niche for the initiation of metastasis (PMN in the liver is illustrated in the figure). **F** Several factors, including exosomes, miRs, and immune cell secreted factors, such as MIF, cytokines, and various chemokines, guide the development of PMN. **G**, **H** Hepatic stellate cells and Kupffer cells initiate fibrosis at the early stages of PMN development, and different chemokines, MMPs, and cytokines facilitate adaptation of tumor cells to the PMN. **I** Liver metastasis with different immune cells and metastasis-associated fibroblasts and resident hepatic cells. Abbreviations: PMN- pre-metastatic niche; MHC- major histocompatibility complex; EMT- epithelial to mesenchymal transition; PD-L1- programmed death receptor ligand-1; MIF- macrophage migration inhibitory factor; miR- micro-RNA; ncRNA- non-coding RNA; TGFβ- tumor growth factor- β; IL- interleukin; MMP- matrix metalloprotease

Most studies investigating the immunological attributes of PDAC suggest that immunosuppression contributes to PDAC progression and poor response to immunotherapy [16, 18]. However, in contrast to its well-studied role in advanced-stage PDAC tumors, the role of immunosuppression in neoplastic initiation, pre-metastatic niche (PMN) development, and organ-specific metastatic growth is poorly understood. In fact, immunoediting, a dynamic process that eliminates the neoplastic cells in cancer, is compromised, which allows tumor cells to survive the immune equilibrium phase and escape the host immunological defense [28, 29]. In the early immunosuppressive niche, the cross-talk between neoplastic cells and their neighboring stromal fibroblasts and regulatory immune cells render them to re-calibrate their roles to promote tumor growth, EMT, and metastasis [18] (Fig. 1). Nonetheless, the dynamic and context-dependent role of immunosuppression, which may vary depending upon PDAC progression stage and its subtype, needs to be assessed when exploiting therapeutic vulnerabilities conferred by immunosuppressive factors [16]. For instance, a recent investigation identified early and late immunosuppression as distinct mechanisms represented by phenotypically and functionally diverse immune cells [13]. In this study, Yang et al. showed that early-stage immunosuppression is conferred by highly abundant regulatory T cells (T-regs) during acinar to ductal metaplasia, while late-stage immunosuppression is predominantly regulated by myeloid-derived suppressor cells (MDSCs). Interestingly, the abundance of effector T cells gradually decreases in the dynamically changing immunosuppressive *milieu* from early to late-stage PDAC progression. In contrast, arginase1 (Arg1^+^) monocytes, representing M2-type of tumor associated macrophages (TAMs), are found to be enriched with disease progression, suggesting that early and late-stage PDACs have distinct immunosuppressive characteristics.

Early metastasis is the most common pathological characteristic in PDAC observed at the time of diagnosis, which is associated with poor therapy response and survival of PDAC patients [1, 10]. Thus far, different molecular mechanisms associated with PDAC metastasis have been identified [30, 31], but the understanding about their immunological regulation is limited. More than a century ago, a research group at Harvard University reported that insufficiency of concomitant immunity, the immune response generated against primary neoplastic growth, allows cancer cells to escape the primary tumor site and metastasize to distant organs [32–34]. PDAC tumors, being poorly immunogenic, elicit low concomitant immunity, which might be a reason promoting their early metastases. In addition, both local and systemic immunosuppressive factors contribute to aggravate metastatic progression, primarily by supporting a subset of tumor cells to undergo EMT and facilitate their escape from host immune surveillance at the primary tumor site and survival in the metastatic niche [10, 35]. During early stages of oncogenesis, neoplastic cells are recognized and killed by surveillant effector immune cells to maintain the immune equilibrium in the host. However, a subset of tumor cells attains EMT and an immunosuppressive phenotype by MHC downregulation and poor antigen presentation, which allows their escape from immune cell-mediated killing in the circulation (Fig. 1C). Tumor cells escaping from the primary tumor site adopt lymph-vasculature to migrate to distant organs [30, 36, 37]. There is a report that tumor cells may also disseminate via perineural invasion (PNI), which might be a further mechanism to escape immune surveillance [38].

The preferential metastasis to a particular organ depends on the organ-specific microenvironment, its anatomophysiological characteristics and its immune cell composition. Thus, the immunosuppressive *milieu* at the metastatic site is likely distinct from the site of origin in the context of composition, phenotypes, and functionality of the immune cells [39]. Remarkably, developing a pre-metastatic niche (PMN) is the first step initiated by extracellular vesicles, exosomes, cytokines, and chemokines secreted from the primary tumor site [39, 40]. These messengers customize the niche by recruiting and activating fibroblasts and immune cells prior to the homing of incoming metastatic cancer cells [41–43] (Fig. 1G-I). In PDAC, the liver is the most common metastatic site, followed by the peritoneum, lung, and pleura [44]. The development of the PMN and establishment of early metastasis is regulated by immune cells in the metastatic microenvironment. Recently, a multi-omics analysis of the liver and lung tissues harboring metastatic lesions showed that each metastatic site exhibits unique immune phenotype and immunoregulatory pathways [45]. The baseline immune infiltrate was higher in the lungs than in the liver, even in the absence of metastasis, suggesting that each organ offers different immune privileges. This study used Panc02 cell line for hemispleen and intravenous injections to establish liver and lung metastases, respectively. When analyzed by mass cytometry, lung samples showed higher expression of co-stimulatory (CD69, ICOS, CD27) and co-inhibitory (PD1-PD-L1 axis, KLRG1, and BTLA) molecules compared to the liver samples, which was further validated in corresponding human metastatic PDAC samples. Importantly, LAG3^+^ CD8 T cells and NK cells were enriched in the liver compared to metastatic lung samples, suggesting that the liver constitutes a more immunosuppressive microenvironment compared to the lungs. These findings substantiated the survival data showing that PDAC patients with lung metastasis have better prognoses and overall survival compared to patients with liver and other metastases [46]. Thus, metastatic sites exhibit variability in the degree of immunosuppression and understanding immune cell abundance and their specific temporal contribution to PMN immunosuppression is important to design specific and effective therapeutic approaches.

The recruitment of various immune and stromal cells such as CAFs, stellate cells, metastasis-associated macrophages (MAMs), neutrophils, T-regs, and MDSCs in the PMNs has been shown to facilitate immunosuppressive *milieu* and further development of metastases (Fig. 1H) [36]. Previous studies suggest that immune cells follow sequential waves to patrol and accumulate in the PMNs of different organs, which are different in immune cell composition and function for each PMN. For instance, neutrophils were earlier recognized as the first cell type migrating to the lung PMN, followed by the second wave of local macrophages and circulatory monocytes and dendritic cells [47]. In contrast, immunosuppressive monocytes have been reported as the first cells migrating to, and helping the PMN formation in the liver [48]. Thus, the immune infiltrate and its immunosuppressive characteristics, as determined by sequential waves of infiltrating immune cells, are critical for the establishment of PMNs and can potentially guide their response to immunotherapies in metastatic PDAC. Hepatic macrophages, either resident Kupffer cells (KCs) or recruited from the peripheral blood, due to their inflammatory phenotype play a critical role in the development of PMN and promote liver metastasis [49]. Mechanistically, KCs uptake the signal-carrying exosomes released from the primary tumor site and begin to release TGF-β, which activates hepatic stellate cells (HSCs) to release fibronectin and promote the recruitment of bone marrow derived macrophages and neutrophils in the PMN (Fig. 1G-H) [42, 50]. In addition, macrophages predominantly release cytokines and chemokines such as IL6, IL10, and CCL2 thereby contributing to the immunosuppressive PMN. Another study showed that the recruitment of granulin-secreting inflammatory monocytes activated HSCs into periostin-secreting myofibroblasts, which promote fibrosis to sustain liver metastasis [48]. Among several tumor secretory factors, SDF1 and CCL2 have been implicated in monocyte recruitment and oncogenic progression, suggesting that these secretory immunosuppressive factors are critical for PDAC progression and metastasis [51, 52].

## Immunoregulatory pathways in PDAC

The stroma, consisting of both cellular and acellular components, has been reported to promote immunosuppression [17, 53, 54]. Previous studies have described the immunosuppressive role of different resident or recruited cells at the primary and metastatic sites [27, 35, 55, 56]. Most previous studies investigating immunosuppression in PDAC were focused on the primary tumor rather than the metastatic sites. Moreover, in-depth multi-omics analysis was lacking, possibly due to unavailability of tools that have been recently developed to analyze different aspects of the TME at the single cell level, including single cell and spatial transcriptomics, mass cytometry (CyTOF), secretome analysis arrays, and high throughput computational tools [57–62]. Despite these limitations, previous efforts have delineated different molecular pathways regulating immunosuppression and their role in PDAC metastasis. Neurogenic locus notch homolog protein 1 (Notch), Hippo, signal transducer and activators of transcription (STATs), IL10, and transforming growth factor-β (TGF-β), Wnt, and hypoxia inducible factor (HIF)-regulated pathways have been implicated in promoting both immunosuppression and metastasis in PDAC [63–66]. For example, STAT-regulated pathways are activated in immunosuppressive TAMs, CAFs, and MDSCs in PDAC [66–68]. In transcriptome profiling of patient-derived MDSCs, STAT3 expression correlated with monocyte reprogramming and immunosuppression, and STAT3-expressing monocytes regulated the arrest of T cell proliferation. Moreover, treatment with a small molecule inhibitor of STAT3 showed abrogation of suppressive activity of CD14^+^ cells, suggesting a direct role of STAT3 in MDSC-mediated immunosuppression. Notably, this study showed that immunosuppressive MDSCs are more abundant in the peripheral blood of PDAC patients that are characterized by a STAT3^+^/Arg1^+^ phenotype of CD14^+^ immune cells and correlate with poor survival of PDAC patients [69]. However, another study indicated that CD15^+^ but not CD14^+^ MDSCs expressing the immunosuppressive receptor CD200 play a predominant role in MDSC-mediated immunosuppression [70]. Furthermore, CAF-heterogeneity in pancreatic TME has been instrumental in driving immunosuppression and potentiating metastatic progression, besides its role in desmoplasia [27, 71]. A recent study showed that CAF-specific inhibition of STAT3 diminished fibrosis and suppressive F480^+^CD206^+^ M2 macrophages, thereby increasing the effector CD8^+^ T cells [68]. Although this study was not followed up to evaluate the effect on metastatic progression, it is likely that STAT3-mediated fibrosis and immunosuppression promote metastatic progression in PDAC.

Activated Notch has been shown to be critical in inducing immunosuppression in PDAC, primarily by regulating myeloid cells [72]. A recent study showed that inhibiting Notch signaling upregulated PD-L1 in PDAC cell lines [65], suggesting that Notch inhibition may sensitize PDAC tumors for anti-PD-L1 therapy. The combination of a Notch inhibitor with anti-PD-L1 therapy resulted in a significant increase in CD8^+^ T cell infiltration and reduction in Ki67 staining, which cumulatively reduced immunosuppression and improved anti-tumor efficacy. Considering the effect of Notch signaling on PD1-PD-L1 axis upregulation, a more recent study revealed that a γ-secretase inhibitor (GSI) targeted the Notch pathway and exhibited strong synergistic anti-tumor effect with anti-PD1 therapy [72]. At a cellular level, this study showed that M2-TAMs expressing high Arginase 1 (Arg1) express high levels of Notch receptor, and targeting Notch signaling reduced immunosuppressive markers such as Arg1, TGFβ, and IL10 in PDAC tumors. Collectively, these studies show that Notch signaling and its collaborative role with the PD1-PD-L1 pathway contribute to immunosuppression in PDAC. However, it will be interesting to investigate further whether this axis contributes to immunosuppression in the PMN and promotes metastatic progression in PDAC.

In addition to the molecular pathways, pathophysiological factors in the pancreatic TME, such as hypoxia, play an essential role in PDAC progression, immunosuppression, metastasis, and immunotherapy resistance [73–76]. Recent evidence suggests that hypoxia-inducible factors (HIFs) are primarily associated with M2-type TAM polarization, and type-2 innate lymphoid cell (ILC)-mediated immunosuppression [77–79]. Ye et al. performed serum biomarker analysis showing that triple biomarker positive PDAC patients [CEA^+^/CA125^+^/CA19-9^+^ levels > 1000U/mL] have a HIF-1α-induced immunosuppressive TME, which promotes the transformation of type 2 ILCs into regulatory ILCs. These regulatory ILCs have been reported to promote immunosuppression in PDAC [79]. On the other hand, Garcia et al. showed that CAF-specific HIF-2α, but not HIF-1α, plays a major role in promoting immunosuppression via increased recruitment of M2-TAMs and Tregs in the spontaneous mouse model of PDAC [78]. In addition, this study highlighted that the deletion of HIF-2α also reduced fibrosis, suggesting a summative role of HIF-2α in immunosuppression and fibrosis. Notably, increased hypoxia in PDAC tumors results from poor vascularity and disrupted lymphatic drainage, which leads to elevated physical stress, and compromised removal of metabolic waste [76, 80]. The hypoxic *milieu* alters the metabolic characteristics of all resident cells that helps cancer cells in immune evasion and metastasis. However, hypoxia is not a significant factor in the PMNs and metastatic microenvironment, unlike primary tumors. Therefore, the therapeutic relevance of hypoxia-associated factors at metastatic sites has been understudied in the context of immunosuppression and immunotherapy.

## Immunosuppression in metastatic PDAC

Distant metastases are largely untreatable and remain the primary factor contributing to the poorest survival of PDAC patients [1]. However, growing evidence suggests that targeting metastasis can improve survival in PDAC patients [81, 82]. Unfortunately, most immunotherapy trials performed in advanced and metastatic PDAC patients have failed to improve clinical outcomes [83, 84]. Possible reasons for the failure of immunotherapies could be 1) poor immunogenicity, 2) heterogeneity at primary and metastatic microenvironments, and 3) increased immunosuppression and resistance to immunotherapy. Efforts have been directed to enhance tumor immunogenicity and mitigate immunosuppression and immunological resistance in PDAC [12, 83]. However, a limited understanding of the immunological attributes of metastatic progression has made it challenging to inhibit or reverse immunosuppression in metastatic PDAC.

Considering metastasis as a sequential series of events, it is believed that there are three distinct sites to explore immunosuppressive pathways: primary tumor site-where immunosuppression promotes EMT and a metastatic phenotype in the disseminating cells; the peripheral blood- which facilitates the dissemination of circulating tumor cells to different organs; and, the target metastatic organ- where circulatory tumor cells acclimatize in the PMNs after intravasation and eventually inflict the metastatic burden. The immunosuppressive *milieu* is mostly defined for the primary tumor site. However, recent studies have also identified different TME factors and molecular triggers that play a significant role in the initiation of metastasis locally and its progression to distant organs. Several immunosuppressive factors present in tumor secretome, such as exosomes, cytokines and chemokines, non-coding RNAs, microbiome, and metabolites, are critical to induce immunosuppression and metastasis in PDAC. In addition, oncogenic pathways driven by activated kinases and alterations in cellular metabolism within the tumor and metastatic microenvironments play a critical role in inducing immunosuppression and establishing distant metastasis in PDAC. The following sub-sections summarize various secretory, molecular, and metabolic factors contributing to the immunosuppression in metastatic PDAC.

### The immunosuppressive secretome

Previous studies have identified various stromal factors such as hypoxia, fibrosis, and suppressive secretome released from the stromal cells that induce stemness, EMT, and metastasis in the tumor cells [10, 80]. Specifically, CXCRs, IL6, TGFβ, the Wnt/Snail, and altered metabolic pathways, hypoxia, hematopoietic cell kinase (HCK), MYC, and complement signaling, have been reported to potentiate metastasis and disease relapse in PDAC (Fig. 1C) [64, 85–88]. Notably, the immunosuppressive role of IL6 and TGFβ cytokines has been extensively studied in metastatic PDAC [89, 90]. In contrast, other cytokines such as, IL1β, IL17, IL18, and TIMP1 (more recognized as a metalloprotease), have also been found to promote immunosuppression and metastasis in PDAC [91–93], suggesting that cytokines play disticnt roles in metastatic progression in a context-dependent manner. Interestingly, the pro-inflammatory cytokine IL6 is upregulated in PDAC patients and recognized for its pro-invasive role in PDAC models. For instance, IL6 stimulation was found to activate small GTPase CDC42, promoting pre-migratory filopodia formation in cancer cells [89]. This activation of IL6-mediated CDC42 activation is regulated by JAK2/STAT3 signaling. Another study showed that IL6, together with leukemia inhibitory factor (LIF) inhibited the tumor suppressor transfer RNA-derived fragment-21 (tRF-21), which is associated with PDAC metastasis as reduced tRF-21 expression correlated with high metastatic burden and poor survival [94].

The master regulator of immunosuppression, TGFβ, is the most validated immunosuppressive cytokine regulating PDAC invasion and metastasis. Several investigations have demonstrated that TGFβ promotes invasion and metastasis, primarily through SMAD family members, VEGF, ICAM1, and microRNAs [95–102]. TGFβ enhanced the VEGF-induced angiogenesis and simultaneously reduced the pancreatic tumor immunogenicity, leading to increased liver metastasis [102]. Elevated TGFβ1 was shown to reduce the intercellular adhesion molecule-1 (ICAM1) expression in PDAC cells causing decreased adhesion to peripheral blood mononuclear lymphocytes (PBMLs) and diminished cytotoxicity on co-cultured PDAC cell lines, suggesting a role of TGFβ1 in cell adhesion and immunotoxicity. In addition, pretreatment with TGFβ1 potentiated liver metastasis when CAPAN-2 cells were injected via the splenic route in mice [101], further providing evidence to support the role of TGFβ1 in metastatic progression. SMAD pathways are found critical to TGFβ mediated immunosuppression and metastasis [103]. Recently, TNF-superfamily-9 (TNFSF9) was reported to regulate the release of IL10 and TGFβ that play a crucial role in immunosuppression and migration of PDAC cells [86]. Mechanistically, TNFSF9 activated Wnt/Snail signaling, further promoted M2-type macrophage polarization, promoted immunosuppression, and enhanced metastasis. Previously, the small GTPase Rac1b was found to negatively regulate TGFβ-induced metastatic characteristics of PDAC cells via modulation of SMAD3 pathway [99]. Recently, the Frizzled receptor of Wnt signaling, FZD7, was reported to potentiate EMT and stemness, leading to enhanced hepatic metastasis of PDAC. The pro-metastatic role of FZD7 was regulated by TGFβ/SMAD3 signaling [95]. Conversely, integrin alpha 2 (ITGA2) overexpression decreased TGFβ-mediated SMAD2-signaling and its impact on metastatic progression.

Several studies have demonstrated pro- and anti-metastatic functions of microRNAs (miRs) in regulating TGFβ-mediated metastasis in PDAC. For instance, miR-10b inhibited Tat interacting protein 30 (TIP30) and promoted EGF and TGFβ-induced invasive properties of PDAC cells [104]. Similarly, miR-323-3p targets SMAD2 and SMAD3, which are downstream mediators of TGFβ. Interestingly, knockdown of miR-323-3p in a mouse model resulted in a significant reduction in lung metastasis of PANC1 cells [98]. Later, other miRs such as miR-23a, miR-193a, and miR-501-3p were demonstrated to stimulate PDAC metastasis via selective TGFβ-receptor signaling [105–107]. More recently, miR-145 was reported to be downregulated in PDAC, and restoration of its expression inhibited TGFβ signaling and reduced EMT, stemness, and metastatic properties in PDAC cells [100].

Various chemokines/chemokine axes, including CXCR3-CXCL9/10/11, CXCL12/stromal cell-derived factor-1 (SDF-1)-CXCR4, CCL21/CCR7, CCL5/CCR5, CXCL8/CXCR2, and CX3CL1/CX3CR1, have been shown to play a vital role in PDAC metastasis [108–113]. PDAC patients with high CXCR4 have poor overall survival, and elevated expression of CXCR4 is associated with PDAC metastasis [108, 109]. Moreover, the effect of high CXCR4 expression on metastatic phenotype is mediated by the Wnt/β-catenin pathway and is associated with increased expression of EMT markers such as vimentin and slug. Inhibition of the CXCL12-CXCR4 axis with a CXCR4 inhibitor was shown to reduce the metastatic potential of MIA PaCa-2 cells [114]. Interestingly, this study showed that CXCL12-CXCR4 signaling activated matrix metalloproteases MMP2 and MMP9, which enhanced the invasiveness of PDAC cells. However, another study showed the tumor-suppressive role of CXCL12 in PDAC using the gene-silencing model [115]. CXCL12 was demonstrated to play a biphasic role in regulating metastasis and bioenergetic homeostasis [116]. Low chemokine concentrations elicited chemotaxis, whereas higher CXCL12 concentrations reduced chemotactic migration. In addition, high CXCL12 promoted CXCR4-dependent myosin light chain phosphorylation, which is required to maintain bioenergetic homeostasis in cancer cells. In the context of immunosuppression, CXCR3, CXCR2, CXCR4, and CCR7 mediated signaling cascades were reported to play predominant roles in immune evasion, immunosuppression, and immunotherapy resistance [112, 117–120], linking chemokine signaling to metastasis and immunosuppression. Thus, both cytokine and chemokine signaling are critical for driving immunosuppression and metastasis and selective targeting of these soluble factors could alleviate the immunosuppression and metastasis in PDAC (Table 1).

**Table 1:** Summary of clinical trials targeting immunosuppression in combination with ICB therapy

**S. No.**	**Target**	**Expression**	**inhibitor**	**Combination immunotherapy**	**PDAC Stage**	**Phase**	**Clinical outcome**	**References**
1	TGFβR1/2	CAFs, TAMs, and other immune cells	M7824: PD-L1 Ab fused with TGFβR2-ECD trap	Fusion Monotherapy	Locally advanced PDAC (*n* = 19 patients)	Phase I	Indications of durable partial response; manageable safety profile	NCT02517398 [121]
2	TGFβR1		Galunisertib (LY2157299)	Durvalumab (PD-L1)	Refractory PC (*n* = 37 patients)	Phase Ib	1 patient had partial response, 7 patients’ stable disease: and 15 patients’ objective progressive disease. Disease control rate 25%. Median OS = 5.72 months.	NCT02734160 [122]
3	TGFβ2		BCA101: anti-EGFR Ab fused with TGFβR2-ECD	Pembrolizumab (PD1)	Advanced disease refractory to SOC therapies (*n* = 7 patients)	Phase I	Well tolerated and clinically active as a single agent and with anti-PD1 therapy	NCT04429542 [123]
4	TGFβ2		SHR-1701: PD-L1 Ab fused with TGFβR2 ECD	Fusion Monotherapy	Metastatic/ Locally Advanced PDAC (*n* = 10 patients)	Phase I	2 out of 10 patients showed stable disease: manageable safety profile	NCT03710265 [124]
5	CSF-1R	TAMs	Cabiralizumab (IgG4 hu-mAb)	Nivolumab (PD1)	Advanced/metastatic PDAC	Phase II	Monocyte depletion but no significant clinical benefit	NCT03336216 [125, 126]
6	CSF-1R		AMG-820 (IgG2 hu-mAb)	Pembrolizumab (PD1)	Advanced/metastatic PDAC (*n* = 116 patients)	Phase Ib/II	Immune-related PR in 3 patients and immune-related stable disease in 34 patients	NCT02713529 [127]
7	CSF1		lacnotuzumab	Spartalizumab (PD1)	Advanced/metastatic PDAC (*n* = 13 patients)	phase Ib/II	Well tolerated; Preliminary anti-tumor activity; Disease control rate = 6/13 PDAC patients	NCT02807844 [128]
8	CSF-1R		exidartinib (PLX3397)	Durvalumab (PD-L1)	Advanced/metastatic PDAC (*n* = 19 patients)	Phase I	Clinical benefit rate at 2 months was 21% in the dose escalation cohort; no unexpected toxicity	NCT02777710 [129]
9	CD40	APCs (DCs), myeloid and B cells	APX005M (sotigalimab)	Nivolumab (PD1) ± Chemo	Metastatic PDAC (*n* = 30 patients)	Phase I	Tolerable regimen with 58% PR and 30% SD.	NCT03214250 [130]
10	CD40		APX005M (sotigalimab)	Nivolumab (PD1) ± Chemo	First line Metastatic PDAC (*n* = 99 patients)	Phase II	APX005M + Nivolumab and chemo showed OS = 41.3% with median OS = 10.1 mo. The triple combination provided immune markers associated with survival.	NCT03214250 [131]
11	CD40		CDX-1140	Pembrolizumab (PD1)	Advanced solid malignancies	Phase I	Objective was to find MTD (1.5 mg/Kg)	NCT03329950 [132]
12	CXCR4	Tumor cells, macrophages and MDSCs	BL-8040	Pembrolizumab (PD1)	Chemotherapy-resistant PDAC (*n* = 37 patients)	Phase I	Disease control rate (34.5%); and nine patients with SD, and one patient with partial response.	NCT02826486 [133]
13	CXCR4		BL-8040	Pembrolizumab (PD1)	Recurrent and metastatic PDAC (*n* = 18 patients)	Phase IIb	Results awaited	NCT02907099 [134]
14	CXCR4		LY2510924	Durvalumab (PD-L1)	Previously treated advanced and metastatic PDAC	Phase I	Disease control rate of 37.5% achieved.	NCT02737072 [135]
15	CXCR4		BMS-936564/MDX1338 (Ulocuplumab)	Nivolumab (PD1)	Previously treated advanced and metastatic PDAC	Phase I/II	Terminated with no clinical outcome	NCT02472977 [135]
16	CXCR2	Neutrophils, macrophage, cancer cells, and ECs	AZD5069	Durvalumab (PD-L1)	Previously treated metastatic PDAC (*n* = 23 patients)	Phase II	Well-tolerated regimen with manageable toxicity	NCT02583477 [136]
17	CXCL12	Multiple TME compartments	NOX-A12	Pembrolizumab (PD1)	Pretreated advanced PDAC (*n* = 9 patients)		Induction of immune response and stable disease in 25% patients, with a consistent safety profile in metastatic microsatellite stable (MSS) patients	NCT03168139; [137]
18	FAK	Cancer cells	Defactinib	Pembrolizumab (PD1) ± Chemo	Refractory PDAC (*n* = 17 patients)	Phase I	Increased CD8 + T cell infiltration, but no PR or CR observed	NCT02546531 [138]

*Abbreviations*: *TGFβ1/2R* tumor growth factorβ 1/2 receptor, *CD40* cluster of differentiation 40, *CSF1R* colony stimulating factor1 receptor, *CXCR* chemokine receptor, *CXCL* chemokine ligand, *FAK* focal adhesion kinase, *SOC* standard of care

In addition to soluble factors such as cytokines and chemokines, stroma-secreted collagens, proteases, and other ECM proteins promote immunosuppression and pro-metastatic characteristics in PDAC. For example, collagen-1 (Col-1), a component of the ECM, enhances the metastatic potential of PDAC cells, regulated by several pathways, including c-Jun N-terminal kinase (JNK), phosphoinositide 3-kinases (PI3Ks) signaling, and SIP1-mediated E-cadherin downregulation [139–141]. Particularly, the interaction between cancer cells and collagen was found to be influenced by PIK3 signaling, as depletion of PIK3CB reduced cell adhesion [141]. Similarly, a recent study found that collagen IV contributes to PDAC metastasis as the metastatic nodules showed stromal cell-derived collagen IV depositions in the metastatic niche [142]. Knowledge about the role of these collagens in immunosuppression is limited. However, a recent study using dual recombinase genetic mouse models of spontaneous PDAC showed that depletion of Col-1 from activated stellate cells increased Cxcl5 and enhanced immunosuppression by recruiting MDSCs and decreasing CD8^+^ T cells [143]. Further investigation showed that the Col-1 homotrimer plays an oncogenic role as depletion of Col-1 homotrimer inhibited immunosuppression and PDAC progression [144]. TME proteases also play an essential role in PDAC metastasis, with matrix metalloproteases (MMPs) such as MMP1-3, 9, and 13 being particularly critical. While the role of MMP9 in enhancing PDAC metastasis is well studied [145–147], MMP2, MMP7, and MMP13 have also been implicated [148–152]. However, the role of MMPs in regulating immunosuppression during PDAC metastasis is not well established. A previous study showed that MMP9 plays an essential role in NK cell dysfunction and immunosuppression and that targeting MMP9 could reverse the SW1990-induced NK cell phenotype and cytotoxic function in co-culture studies [153]. Treatment of NK-92 cells with an MMP9-blocker enhanced surface expression of NKG2D and secretion of perforin and granzyme B by these cells, suggesting that MMP9 inhibition can reverse the cancer cell-induced immunosuppressive effect and potentiate NK cell effector function. These studies suggest that the non-immune stromal cell secretome contributes to immunosuppression and PDAC metastasis. However, the proposed direct association of a fibrotic tumor secretome with immunosuppression may further strengthen our understanding of the role of the stromal secretome in immunosuppression and PDAC metastasis.

### Exosomal guidance for PMN formation and metastasis

The RNA and proteins carrying exosomes have emerged as critical mediators of metastatic progression due to their ability to shape PMN formation and metastatic growth [154]. Previous studies have summarized that exosomes are crucial for tumor progression, metastasis, immune evasion, and therapy resistance [155–157]. Although not fully understood, evidence suggests that exosomes carry information to prime distant organs to initiate PMN development (Fig. 1F). Earlier studies demonstrated that PDAC-secreted exosomes regulate the formation of PMNs in the liver, the most common site for PDAC metastasis [158, 159]. Particularly, tumor-derived exosomes were reported to educate hepatic stellate cells (HSCs) and Kupffer cells (KCs) to secrete fibronectin and transforming growth factor-β, respectively, thereby creating a fibrotic and immunosuppressive microenvironment at the metastatic site [160, 161]. These secreted factors help to recruit bone marrow-derived macrophages to hepatic PMNs (Fig. 1F). Interestingly, the macrophage migration inhibitory factor (MIF) was found to be upregulated in exosomal fractions of patients with early-stage PDAC who later developed liver metastases and in a PDAC mouse model of liver metastasis. Moreover, the depletion of MIF led to a significant reduction in PMNs, suggesting a pivotal role of exosome-derived MIFs in promoting a fibrotic and immunosuppressive microenvironment in hepatic PMNs. Further analysis suggested that treatment of HSCs with tumor-derived exosomes promoted membrane transport of complement C1q binding protein (C1QBP) and CD44v6 complex, mediated by insulin growth factor-1. Retrospective analysis of PDAC patients who developed liver metastases after surgery revealed a higher expression of C1QBP and CD44v6 in metastatic lesions and in circulating exosomes. In addition, high expression of the C1QBP/CD44v6 complex associated with poor prognosis of PDAC patients with metastases. Altogether, the C1QBP/CD44v6 complex appears to be critical for the development of hepatic PMNs. Furthermore, tumor-derived exosomal micro-RNAs (miRs) have been reported to play a significant role in metastasis. For example, pancreatic stellate cell-derived exosomal miR-21 promotes EMT and metastasis by targeting Ras/ERK pathway [162]. Tumor-secreted exosomes were also found to contain miR-222, which induced p27 phosphorylation and promoted invasion and metastasis of PDAC [163]. Another study showed that exosomal miR-338 is critical in regulating lymphatic metastasis of PDAC, which is primarily driven by circular RNA, regulating growth and invasion by activating MET/ERK or AKT pathways [164]. In addition, exosomes carrying long non-coding RNAs ln-Sox2ot were found to enhance EMT, stemness, and metastasis in PDAC, by competitively binding to miR-200 and regulating Sox2 expression [165]. More recently, tumor-derived extracellular vesicles and particles (EVP), such as exosomes and exomeres, have been demonstrated to mediate metabolic reprogramming in the liver, causing fatty liver and attenuation of drug metabolism, leading to enhanced side effects, such as bone marrow suppression and cardiotoxicity [166]. Mechanistically, small GTPase Rab27a is found to play a role in EVP secretion and regulate metabolic reprogramming in the liver in a TNF-dependent manner. The TNF release by KCs generated a pro-inflammatory microenvironment and suppressed cytochrome p450 release, fatty acid metabolism, and oxidative phosphorylation. Thus, exosomal guidance is critical to reprogramming of liver metabolism and establishment of liver metastasis in PDAC.

Besides the liver, few reports demonstrate the role of exosomes in metastatic PDAC progression to other organs. Recently, Ogawa et. al. showed that Notch signaling enables pro-metastatic secretome trafficking in lung metastasis [167]. Using in vitro and in vivo models, it was shown that aspartate-β-hydroxylase activates the Notch cascade, which subsequently promotes exosome trafficking and MMP-mediated ECM remodeling for metastatic progression. Thus, exosomes are important regulators during PDAC metastasis. However, further investigations are warranted to analyze the impact of these exosomal payloads and molecular pathways on immunosuppression during PMN development. Similarly, macrophage-derived exosomes have been shown to promote PDAC metastasis [105, 168, 169]. For example, macrophage-derived exosomal miR-501-3p was reported to induce invasion and metastasis by targeting the TGFβR3 [105]. Thus, it is likely that immune cells recruited to the PMNs release exosomes capable of potentiating immunosuppression in metastatic microenvironments. Altogether, targeting exosome-regulated pathways in the PMNs can alleviate immunosuppression and delay metastatic progression in PDAC.

### Non-coding RNAs in metastasis and immune regulation

Non-coding RNAs (ncRNAs), constituting up to 98% of transcriptome, have emerged as significant players in the pathophysiology of different diseases, including cancer, and are considered important targets for the diagnosis and therapy [170–173]. The ncRNAs are classified as long (> 200 nucleotides) and short (< 50 nucleotides) ncRNAs based on their length. Among different ncRNAs, micro-RNAs (miRs) and long-ncRNA (lncRNAs) are the most characterized ncRNAs, while other ncRNAs such as transfer RNA (tRNA)-derived small RNAs (tsRNAs), PIWI-interacting RNAs (piRNAs), circular RNAs (circRNAs), and pseudogenes, are gradually gaining attention for their emerging roles in cancer pathogenesis [174, 175]. Functionally, the miRs bind to the mRNA transcripts and modulate protein translation and their function, while lncRNAs support small RNAs such as miRs by facilitating their binding to the targets and regulate their functions [176]. Recent studies have highlighted the pathophysiological and therapeutic significance of ncRNA in PDAC, highlighting their role in disease progression, metastasis, and therapy resistance [177–180], and their utility as biomarkers and therapeutic targets [181–183]. Interestingly, ncRNAs have been shown to regulate PDAC pathobiology via both tumor-promoting and restraining functions [178, 184]. For instance, recent studies have shown the role of lncRNAs such as TP73-AS1, LINC00842, MALAT1, and LINC00941 in tumor growth and metastasis [185–188], whereas others, such as lncRNA01111 and CASC2 have been demonstrated to suppress PDAC growth [189, 190]. Circular ncRNAs have also been reported to regulate PDAC pathogenesis, primarily by sponging miRs and promoting their functions. For instance, circNEIL3 has been shown to regulate the adenosine deaminase acting on RNA 1 (ADAR1) protein expression by sponging miR-432-5p, thereby regulating tumor progression, EMT, and metastasis [191]. Similarly, circRNT4 promotes EMT and liver metastasis, by binding to tumor suppressor miR-497-5p, which inhibits oncogenic lncRNA HOTTIP and stabilizes the EMT associated Rab11 family interacting protein 1 (RAB11FIP1) [192]. More recently, circSTX6 containing 4–7 exons of syntaxin-6 gene was shown to promote tumor growth and metastasis by sponging miR-449b-5p, which regulates the expression of myosin heavy chain-9 (MYH9) protein [193]. Moreover, circSTX6 regulated HIF-1α in a ubiquitin-dependent manner, suggesting its role in regulating hypoxia in PDAC.

Based on the expression and contribution to various immunoregulatory pathways [194–196], ncRNAs have emerged as important targets for immune modulation and immunotherapy of different cancers, including PDAC [197–200]. However, there is limited evidence to support the role of ncRNAs in PDAC immunosuppression, particularly in regulating immunosuppressive pathways that contribute to metastatic progression. Recent studies have revealed immune cell-associated lncRNA signature correlating with immune infiltrates and disease prognosis [201, 202]. For example, a lncRNA FIRRE showed a positive correlation with CD8^+^ T cell infiltration and patient survival in PDAC. Similarly, the cancer genome atlas (TCGA) database analysis was used to identify EMT-related lncRNA signatures [203]. Interestingly, out of 368 EMT-associated lncRNAs, an eleven lncRNA signature was found to be an independent prognostic factor in segregating low and high-risk PDAC patients. Importantly, EMT-associated lncRNAs showed a high correlation with the immune checkpoint molecules such as PD1/PD-L1 and CTLA4, suggesting an association of these lncRNAs with immunosuppressive characteristics of these PDAC tumors. Similarly, another study reported seven lncRNAs associated with PDAC fibroblasts having high prognostic and immunological significance. This study found a negative correlation between lncRNAs signature and CD8^+^ T cell infiltration in the model scores, suggesting poor immune infiltration in the high-risk group [204]. As genomic instability is one of the characteristics of PDAC, recent studies explored the ncRNA signatures associated with the maintenance of genomic instability and evaluated their prognostic and immunological significance [205]. Interestingly, in one study, genomic instability associated lncRNA signature was found to correlate with the EMT and lower adaptive immunity in PDAC, which was further substantiated in a parallel study showing a strong correlation of genomic instability associated lncRNA signature with CD8^+^ T cells, M1 macrophages, immune checkpoints, and IFNγ in the low-risk cohort compared to the high-risk [205, 206]. The correlation of lncRNA signature with immune checkpoints and other immune parameters suggests their involvement in immune regulation, prognosis, and PDAC immunotherapy.

The ncRNAs regulating molecular pathways associated with immunosuppression and metastasis remain poorly understood in PDAC. However, emerging literature suggests that ncRNAs are the dark horses in regulating molecular, metabolic and immunoregulatory pathways in PDAC. Recently, Glucose transporter 1 (GLUT1) was shown to be a predictor of poor prognosis. The ncRNA CASC19/miR-140-5p, upstream of GLUT1 mRNA, was demonstrated to regulate GLUT1 expression and thereby PDAC progression, metastasis, and immune response [207]. More importantly, high expression of GLUT1 associated with PD-L1, revealing its role in PDAC immunosuppression. The Wnt/β-catenin pathway regulates autophagy and plays an essential role in the immune modulation of cancer [208, 209]. However, the role of ncRNAs in the regulation of the Wnt/β-catenin pathway and autophagy has not been deciphered in the context of immune modulation. In this regard, a recent study has predicted six lncRNAs associated with the Wnt/β-catenin pathway and autophagy, which correlated with immune response in PDAC [210]. Particularly, this lncRNAs signature correlated with high CD8^+^ T cell and M0 macrophage infiltration in the tumors of the low-risk group, suggesting its high prognostic significance. Another study supporting the immunosuppressive role of circular ncRNA in PDAC showed that forced expression of hsa_circ_0046523 tumor cells increased their proliferation and metastatic properties via miR148a-3p [211]. When PBMCs derived from healthy donors were co-cultured with hsa_circ_0046523 overexpressing cells, a significant decrease in effector CD8^+^ T cell population and IFNγ production was observed. In contrast, there was a notable increase in the Tregs in co-cultured PBMCs, suggesting the immunosuppressive role of hsa_circ_0046523 in PDAC. In another study, Sun et al. have identified a lncRNA PVT1 (plasmacytoma variant translocation 1) expressed by the tumor-associated nonmyelinated Schwann cells (TASc) in PDAC tumors as orchestrators of immunosuppression [212]. PVT1 expression in TASc was induced by IL-6 and in turn functionally promoted increased production of immunosuppressive metabolite kynurenine by physically interacting with and augmenting the activity of tryptophan 2,3-dioxygenase (TDO2). More importantly, the depletion of tumor-associated nonmyelinated Schwann cells is by small molecule inhibitor cuprizone improved the efficacy of anti-PD1 therapy. Collectively, ncRNAs are critical to PDAC progression, metastasis, and immunotherapy resistance and have emerged as potential therapeutic targets.

### Kinase signaling in PDAC metastasis

Signaling cascades regulated by various kinases not only potentiate tumor cell proliferation and aggressiveness, but also promote immunosuppression at both primary and metastatic sites (Fig. 1D) [55, 213]. Several kinases, including receptor-interacting serine/threonine protein kinase 1 (RIPK1 kinase), hematopoietic cell kinase (HCK), pyruvate kinase M2 (PKM2), focal adhesion kinase (FAK), and PI3-kinase, have been found to promote immunosuppression and metastasis in PDAC [214–217]. RIP1 plays a major role in TAM reprogramming towards an immunosuppressive M2-subtype and a reduces cytotoxic T-cell response [218]. In addition, pharmacological inhibitor (GSK’547) of RIP1 was demonstrated to alter the phenotype of TAMs from immunosuppressive to immunogenic (MHCII^hi^TNFα^+^IFNγ^+^) with significant reduction in tumor burden and metastasis. Another study showed that PKM2 regulates PD-L1 expression in PDAC cells and contributes to immunosuppression. TAM-released TGFβ1 facilitated PKM2 translocation to the nucleus and transactivate PD-L1. Interestingly, both RIP1 and PKM2 have been reported to induce immunosuppression in a STAT1-dependent manner, and M2-TAMs are critical for inducing immunosuppression and metastasis in PDAC [217, 218]. However, the impact of these kinases at metastatic sites remains to be investigated. Recently, focal adhesion kinase (FAK) has emerged as another important molecular target that plays a role in stromal complexity, metastasis, and immune evasion [219], particularly in PDAC which is highly fibrotic and immunosuppressive [220, 221]. In fact, FAK is highly expressed in pancreatic tumors, and elevated FAK activity was found to induce fibrosis, poor CD8^+^ T cell infiltration, and increased immunosuppressive cells in the TME [221]. Similarly, FAK upregulation, associated with increased Col-1, has been found to increase stemness and therapy resistance in PDAC [222]. To mitigate pro-tumorigenic FAK activity, small molecule inhibitors targeting FAK have been evaluated for reducing fibrosis, immune modulation, and improve therapy response, particularly in the context of ICB therapies that are poorly effective as single agents in PDAC (Table 1) [222–225].

Non-receptor tyrosine kinases such as HCK and Steroid receptor co-activator (SRC) kinase are also key mediators of innate immunity and have been reported to regulate immunosuppression in multiple cancers, including PDAC [67, 213, 226]. Recently, Poh et al. showed in a mouse model that genetic ablation of HCK impaired tumor growth and metastasis [227]. Using a splenic model of liver metastasis, this study demonstrated that the genetic ablation of HCK reduces immunosuppression, metastatic burden, and resistance to immunotherapy. The stem cell marker DCLK1 (doublecortin-like kinase 1) has also been recently identified as a promising kinase involved in cancer stemness, EMT, metastasis, and immune regulation [228]. Using gain-of-function studies, it was reported that both long and short-DCLK1 isoforms are instrumental in inducing EMT to promote metastasis in PDAC. More importantly, this study showed that DCLK1 overexpressing PDAC tumors had decreased T-cell and increased M2 macrophages, suggesting that DCLK1 may regulate immunosuppression. Altogether, several kinase-regulated pathways implicated in the proliferation, metastasis, and immunosuppression have been identified in PDAC and are attractive targets for therapeutic intervention in combination therapies.

### Microbiome in PDAC immunosuppression

The altered microbiome generates inflammatory and immune-modulatory responses and has emerged as a crucial regulator of PDAC progression, metastasis, and therapeutic resistance [229]. In addition to the pathogenic contribution due to gut dysbiosis, pancreatic microbiota has been reported to influence organ-specific inflammatory responses, immunosuppression, and patient survival [229, 230]. It has been previously recognized that the cancerous pancreas harbors more microbiome, distinct from the normal pancreas, and ablation of this pathogenic microbiome protects from preinvasive and invasive PDAC, mainly by reversing tolerogenic PDAC immune TME [231]. This study highlighted that ablation of pathogenic microbiome allows immunogenic reprogramming, predominantly by promoting Th1 differentiation, M1-macrophage differentiation, and reducing MDSCs. Thus, reconstituting the healthy gut/pancreatic microbiome can mitigate immunosuppression in PDAC. To demonstrate the immunomodulatory role of gut microbiome in cancer pathogenesis, Sethi et al. elegantly depleted the microbiome by oral antibiotic treatment in different murine models of PDAC, colon cancer, and melanoma to analyze the immune response [232]. Depletion of the gut microbiome decreased tumor growth and metastatic outgrowth in all the experimental murine models. The anti-tumor effect of gut microbiome depletion was predominantly Th1-mediated and observed in immunocompetent mice but not in T- and B-cell lacking Rag1^−/−^ mice, suggesting that microbiome plays a crucial role in regulating adaptive immune response.

Another study reported a differential intra-tumoral microbiome composition in the long and short-term surviving PDAC patients, with a higher alpha-diversity associated with long-term survival [233]. Microbial signature composed of *Pseudoxanthomonas, Streptomyces, Saccharopolyspora,* and *Bacillus clausii* exhibited high predictive value for long-term survival. Interestingly, fecal microbiota transplantation (FMT) from long and short-term surviving human PDAC patients to syngeneic KPC implantation model showed differential anti-tumor effects. Remarkably, the FMT from long-term survivors enhanced the CD8^+^IFNg^+^ T cell infiltration, reduced FoxP3^+^ Tregs and Ly6G/Ly6C^+^ MDSCs, and reduced tumor growth in the recipient mice. This study suggested that reconstituting healthy microbiome is vital to mitigate immunosuppression and improve survival in PDAC. Considering the differences in the human and murine gut microbiomes, it would be more informative to implant the intra-species microbiota and analyze therapeutic implications of FMT in PDAC immunotherapy.. More recently, Ghaddar et al. developed a method to recover and denoise the single-cell sequencing data to identify tumor-microbiome interactions [234]. This study analyzed two PDAC patient cohorts and reported that somatic cell-associated bacteria was exclusive to the tumor subsets, while there was a negligible presence in the normal pancreas. In addition, the abundance of these bacteria was associated with genes regulating cell motility and immune signaling, suggesting that the pathogenic microbiome modulate metastatic properties of cancer cells and the immune pathways to support PDAC. Metabolomic screening identified immunomodulatory microbiome-released metabolites [235]. This study identified an immunologically relevant metabolite, trimethyl N-oxidase (TMAO), that, when administered intraperitoneally or given as a diet supplement, reversed TAM phenotype, enhanced IFNγ-dependent T-effector function, and improved anti-PD1/anti-TIM3 targeted immunotherapy responses in PDAC murine models. Other microbiome-release metabolites such as butyrate, 3-Indole-acetic acid (3-IAA), and other tryptophan-derived metabolites (discussed in the next section) have been recognized to contribute to PDAC pathogenesis and as potential targets for PDAC therapy. Notably, 3-IAA enhanced the efficacy of PDAC chemotherapy [236]. Altogether, an altered gut microbiome plays a crucial role in PDAC pathogenesis, and depleting microbiome or its metabolites is an emerging therapeutic immune modulatory approach to target PDAC.

### Metabolism and bioenergetics in PDAC

**Fig. 2:**
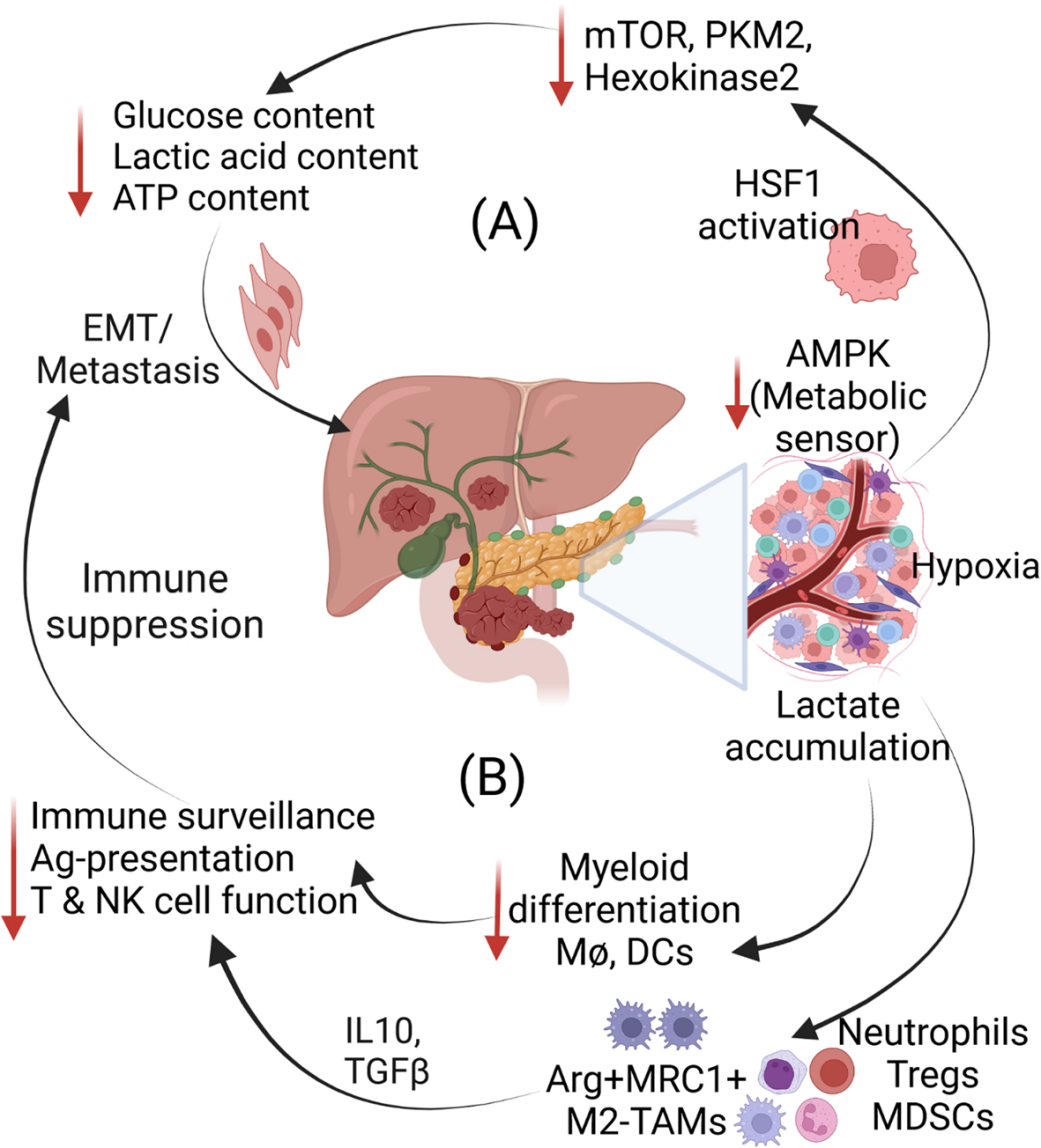
Factors contributing to immunosuppression and PDAC metastasis. **A** Downregulation of AMPK, a metabolic sensor, decreases several metabolic enzymes leading to reduced glucose and ATP levels in the tumor, which triggers cells to undergo EMT. **B** Metabolic priming and lactate accumulation promote the infiltration of suppressive immune cells that contribute to an immunosuppressive cytokine and chemokine pool and deregulate immune surveillance, Ag-presentation, and effector immune functions. Immune dysregulation and increased immunosuppression promote EMT and metastasis in PDAC. Abbreviations: Arg1-arginase 1; MAM-metastasis-associated macrophages MRC1- mannose receptor C-type 1, TAMs- tumor associated macrophages; MDSCs- myeloid-derived suppressor cells; Treg- regulatory T cells; NK cells- natural killer cells; HSF1- heat shock factor -1; mTOR- mammalian target of rapamycin; PKM2- pyruvate kinase M2

Metabolic reprogramming and altered cellular bioenergetics in the PDAC TME contribute to PDAC progression [237, 238]. Thus far, most investigations support the role of altered metabolism and compromised mitochondrial fitness in tumor progression, stemness, and therapy resistance [239–241]. However, in the case of distant metastasis, the stringent mechanisms favoring cancer cells to adapt to the new metabolic microenvironments remain poorly understood (Fig. 1C-D). Within the pancreatic TME, tumor cells undergo clonal selection to survive various genotoxic, metabolic, and immunological stresses. Only a fraction of cells with higher metabolic fitness and immunological adaptability survives during this evolution. Reciprocally, stromal cells such as CAFs, stellate cells, and immune cells mutually interact to reprogram their metabolic needs and establish a metabolic symbiosis in the TME, creating a conducive ecosystem for all the metabolically adapted cells [237]. Cells surviving in the metabolically distinct TME thus change their metabolic sensing, nutrient uptake, and intracellular catabolic pathways, guided by metabolic enzymes and intracellular kinases [242, 243]. For example, adenosine monophosphate-activated protein kinase (AMPK), a well-known energy sensor that plays an essential role in cellular homeostasis, is downregulated during cancer progression and metastasis [242]. Loss of AMPK was found to potentiate PDAC invasiveness and metastatic properties, driven by heat shock factor 1 (HSF1) [244]. In contrast, Hu et al. reported that upregulated AMPK correlated with poor survival and inhibition of AMPK-blocked proliferation and migration [245]. In addition, inhibition of AMPK led to reduced ATP and lactic acid levels and glucose consumption rate in PDAC (Fig. 2A). The metabolic alterations induced by AMPK inhibition were further corroborated by decreased expression of glycolytic markers such as mammalian target of rapamycin (mTOR), pyruvate kinase M2 (PKM2), and hexokinase 2 (HK2), suggesting that AMPK is an important molecular target in PDAC.

In response to reduced extracellular nutrient availability, cells slow down the anabolic pathways of protein and lipid biosynthesis and/or activate autophagy to scavenge and recycle nutrients [246]. Several pathways associated with glucose and glutamine biosynthesis, including the hexosamine biosynthesis pathway (HBP) and pentose phosphate pathway (PPP), are altered depending on the nutrient *milieu* in the tumor [237]. Following the more than one century-old “Warburg effect” hypothesis, studies have shown that metabolically active cancer cells compete for available extracellular glucose depriving immune cells, which is the underlying reason for metabolic defects and altered effector functions of the immune cells [246]. Thus far, the effects of altered TME metabolic factors on immune cell metabolism and immunosuppression have not been thoroughly investigated in PDAC. Accumulation of lactate in the TME inhibits monocyte and dendritic cell differentiation and reduces immune surveillance and effector functions of T cells and NK cells [247, 248]. Moreover, high lactate was reported to induce Arg1^+^MRC1^+^ M2 TAMs that secrete high levels of IL10 and promote immunosuppression [249]. Thus, it is assumed that increased immunosuppression and dampened T cell effector function could result from enhanced lactate metabolism in the pancreatic TME (Fig. 2B). Recently, it was reported that sustained lactate release after radiotherapy promoted the pro-tumorigenic role of MDSCs in PDAC, which was mediated by the G-protein coupled receptor 18 (GPR18)/mTOR/HIF-1α pathway. Interestingly, this study highlighted that the anti-tumor T cell response can be reinstated by blocking lactate production or targeting HIF-1α [241].

**Fig. 3:**
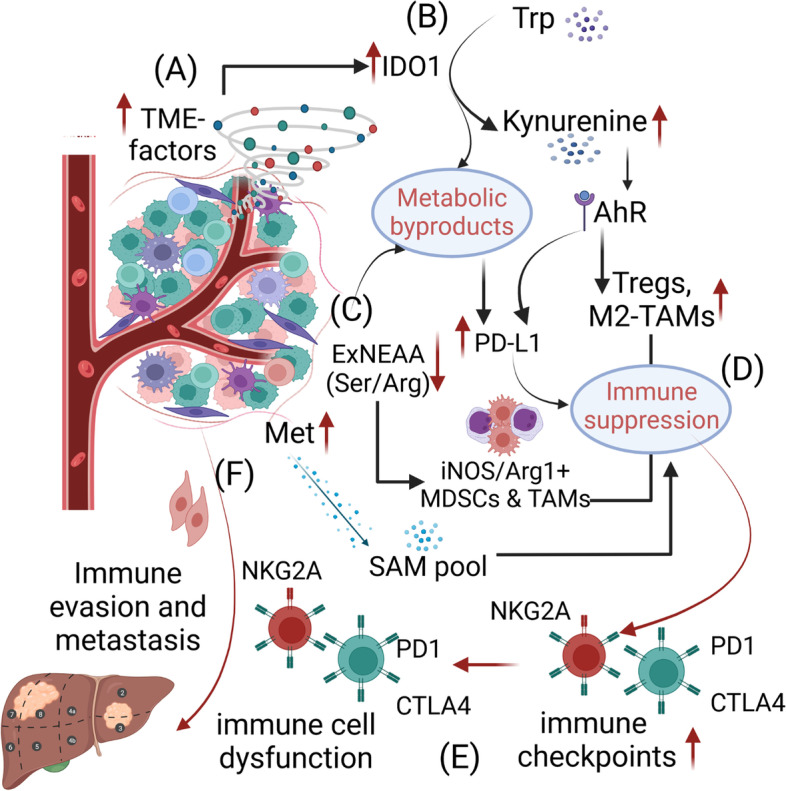
Metabolic alterations regulate immunosuppression in metastatic PDAC. The metabolic microenvironment in pancreatic tumors changes with disease progression. **A**, **B** Altered Trp catabolism due to upregulated IDO1 result in high kynurenine levels which activates AhR to promote enrichment of Tregs and M2-TAMs, resulting in  enhanced immunosuppression in the TME. **C**, **D** Metabolic byproducts and high levels of non-essential AA (serine/arginine) and extracellular methionine enhance immunosuppression in PDAC tumors. **E**, **F** Increased immunosuppression is associated with immune dysfunction, immune evasion, and metastasis. Abbreviations: Trp- tryptophan; AhR-aryl hydrocarbon receptor; IDO1- indoleamine 2, 3-dioxygenase 1; ExNEAA- extracellular non-essential amino acid; PD1- programmed cell death receptor 1; CTLA4- cytotoxic T-lymphocyte antigen-4; iNOS- inducible nitric oxide synthase; Met- methionine; Ser- serine; Arg- arginine; SAM- S-adenosyl methionine

Amino acid catabolism and their extracellular abundance are equally critical in regulating the immunosuppressive TME in PDAC. Previously, tryptophan catabolism and the abundance of other amino acids such as arginine, serine, and methionine, have been reported to play a direct role in immunosuppression (Fig. 3). For instance, altered tryptophan (Trp) catabolism has been reported to regulate immunosuppression [250]. Upregulation of rate-limiting enzyme Indolamine 2,3- dioxygenase-1 (IDO1) promotes the conversion of Trp to kynurenine (Aryl hydrocarbon) which in turn enhances Treg and M2 TAM population in the TME (Fig. 3B). Moreover, increased Trp catabolism in cancer cells releases immunosuppressive metabolic byproducts that upregulate PD-1 on CD8^+^ T cells [250, 251]. Several approaches that inhibit the rate-limiting enzyme IDO1 alleviate immunosuppression and are being extensively evaluated for targeting different cancers, including PDAC [252–255]. The gut microbiome has been reported to play an important role in Trp catabolism and subsequent immunosuppression and tumor growth in PDAC [251]. Particularly, a study highlighted the role of TAM-expressed aryl hydrocarbon receptors (AhRs) in blunting anti-tumor immunity through the upregulation of immune checkpoints and Tregs in PDAC tumors. In contrast, the bacteria in gut microbiome such as *Lactobacillus murinus* were reported to convert the dietary tryptophan to indoles, thereby quenching the substrate for TAM-expressed AhRs, which led to increased intratumoral accumulation of TNF^+^IFN^+^CD8^+^ T cells and increased anti-tumor immune response [251]. Altogether, elevated Trp-catabolism is emerging as an important metabolic vulnerability that can be exploited to alter the regulatory immune phenotype and alleviate immunosuppression in PDAC (Fig. 3B). Similarly, methionine, which plays a major role in protein biosynthesis and methylation, regulates immunosuppression under various pathological conditions, including cancer [256–259]. Reduction of oxidized methionine residues has been reported to suppress metastasis, which was found to be regulated by an enzyme methionine sulfoxide reductase A (MRSA). Loss of MRSA causes selective oxidation of methionine residue M239 in PKM2, promoting PDAC metastasis [257]. Moreover, an abundance of methionine increases the S-adenosyl L-methionine (SAM) pool, a critical substrate for methylation reactions involved in epigenetic modifications, that impacts T-cell phenotype and function and promotes immunosuppression [259]. Similarly, non-essential amino acids (NEAAs) such as serine and arginine have been found to promote immunosuppression (Fig. 3C). Low extracellular arginine and serine increase iNOS^+^/Arg1^+^ MDSCs and TAMs, which directly dampen T cell effector function [260–262]. In addition, sufficient levels of these NEAAs have been shown to enhance the central memory response and function of CD8^+^ T cells. How the immunosuppressive factors triggered by metabolic stress potentiate metastasis is largely unclear. The concerted role of altered metabolism and immunosuppression in developing PMNs and latent or active metastases needs a thorough understanding for identifying the targetable “hot spots” to attenuate the metastatic progression. A previous study identified disseminating cancer cells (DCCs) in PDAC with immunologically distinct phenotype with loss of CK19 and MHC1 expression. These DCCs were thought to be the result of clonal selection after encountering the anti-tumor immune response. Moreover, prolonged ER stress in these DCCs was reported to help escape adaptive immunity and develop latent metastasis [263].

Mitochondria, the powerhouse of the cell, plays a critical role in tumor growth and metastasis. Liang et al. showed that dynamin-related protein-1 (DRP-1) regulates mitochondrial fission, which enhances aerobic glycolysis to promote PDAC progression and metastasis [264]. Compromised mitochondrial fitness profoundly contributes to EMT and metastasis [265]. A recent study suggested that increased mitochondrial redox signaling promotes EMT and metastasis in PDAC, and targeting it with MitoQ, a mitochondria-targeting anti-oxidant, could inhibit metastasis in a murine PDAC model. These studies suggest that targeting altered metabolism and reinstating mitochondrial fitness are potential options to alleviate metastasis. The role of altered immune cell metabolism in promoting immunosuppression and metastasis remains poorly understood. Although cancer cells residing in the altered metabolic microenvironment have been reported to exhibit enhanced stemness, EMT, therapy resistance, and immune evasion, immune cells in the same metabolic microenvironment are thought to exhibit altered phenotypes and functions. Previous studies have suggested that suppressive immune cells such as MDSCs, M2-TAMs, neutrophils, and T-regs are better equipped to cope with metabolic stress in the TME, whereas immune effector cells such as effector T cells, NK cells, and APCs are unable to retain their phenotypic and functional characteristics [18]. A previous study showed that limited glutamine and leucine supply in the TME restricted naive CD4^+^ T cells from differentiating into Th1 and Th17 cells. At the same time, no effect on Treg cells was observed [266]. This effect was mediated by an amino acid transporter, alanine serine cysteine transporter 2 (ASCT2), which is abundantly expressed on naïve CD4^+^ T cells. As glutamine is a limiting metabolite in PDAC, this could be a reason for the low abundance of Th1 cells. In contrast, Tregs are not affected by low glutamine and are therefore more frequently observed in pancreatic TME. Interestingly, studies suggest that neutrophils are the first immune cells to reach and support the PMN formation [87, 267], while macrophages prepare tumor cells for dissemination from primary tumors and establishment in the PMN by inducing liver fibrosis [48]. In contrast to the primary tumors, the metabolic phenotypes of TAMs change during metastasis, right from the initiation of cancer cell dissemination. At the initial steps of metastasis, TAMs produce high levels of nitric oxide (NO), consume less glucose, and upregulate glutamine synthesis, whereas in the PMN, TAMs show NADPH oxidase 1/2 (NOX1/2) deficiency but high glutamine synthesis and lower glucose consumption [268]. These TAMs are Arg1^+^/MRC1^+^ and release high levels of IL10, TGFβ, and several immunosuppressive chemokines that support cancer cells to survive as CTCs and establish the PMN. In addition, the immunosuppressive microenvironment alters the phenotype of effector T cells, leaving them with high expression of immune checkpoints and low release of interferons (IFNs), granzymes, perforins, and reduced memory T cell markers such as the homing receptors L-selectin (CD62L) and chemokine receptor-7 (CCR7). Altogether, altered metabolic programming impacts cancer cells and immune cells and allows them to gain an immunosuppressive phenotype and support tumor progression, metastasis, and resistance to PDAC immunotherapies.

## Immunotherapies targeting PDAC Immunosuppression

Upregulated inhibitory immune checkpoint molecules (ICs) in the tumor and metastatic microenvironments are a major cause of poor response to immunotherapies. Additionally, poor outcomes of PDAC immunotherapies are due to a combined effect of low antigen repertoire, poor antigen presentation, MHC-downregulation, and fibrotic and immunosuppressive TME. In recent years, the discovery of ICs and their targeting have revolutionized the treatment paradigm in certain cancers, including melanoma and non-small cell lung cancer (NSCLC), but have shown a limited efficacy in PDAC [269, 270]. Thus far, several inhibitory IC molecules have been identified that play a vital role in suppressing the anti-tumor immune response generated by NK and T cells, the two main cytotoxic lymphocytes performing anti-tumor activity. The NK cell expresses not only its classically known IC molecules, such as killer immunoglobulin-like receptors (KIRs), leukocyte immunoglobulin-like receptors (LIRs), and NKG2A/CD94, but also recently identified inhibitory molecules, such as B7-H3, CD73, CD96, CD200, and Siglec family members [70, 271, 272]. In addition, NK cells moderately express other IC molecules that are known to be expressed on T-cells, including the programmed cell death receptor-1 (PD-1), T cell immunoglobulin and mucin domain 3 (TIM3), lymphocyte activation protein 3 (LAG3), and T cell immunoreceptor with Ig and ITIM domains (TIGIT). Recent discoveries include other immunosuppressive molecules from the B7-family, including B7-H3 and V-domain Ig suppressor of T cell activation (VISTA), which negatively regulate T cell function [273–275]. Most ligands/receptors binding to these IC molecules are expressed on various cells in the pancreatic TME, including cancer cells, CAFs, TAMs, and MDSCs. For instance, PD-L1 and PD-L2, ligands for PD1, are highly expressed on most cell types in the pancreatic TME [276, 277]. Similarly, LAG3 which binds to MHCII, is expressed on different cell types, predominantly on TAMs and APCs. CTLA4 expressed on T cells binds to CD80/86 with higher affinity than CD28, thereby inhibiting T cell effector function [278, 279]. Approaches targeting immunosuppression have been evaluated with other Immunotherapies targeting ICs, co-stimulatory pathways, and therapeutic vaccines to establish their combined efficacy in PDAC.

### PD1-PD-L1 axis

Despite several studies reporting targetable expression of IC molecules in PDAC, ICB therapies have exhibited limited efficacy, primarily due to stromal complexity and resistance caused by immunosuppressive TME factors [16, 270]. Targeting key immunosuppressive and fibrotic pathways is being actively investigated for improving the outcome of various immunotherapeutic approaches [18, 54, 280]. Immunosuppressive cytokines TGFβ and IL10 upregulate PD1, and blocking signaling pathways regulated by these suppressive factors has improved the response to ICB therapy [281, 282]. Concomitant inhibition of TGFβ and PD-L1 improved the anti-tumor immune response in PDAC. In this study, acidic pH-responsive nanoparticles were used to deliver TGFβR inhibitor together with siRNA targeting PD-L1 in a Panc02 xenograft model. Interestingly, inhibition of TGFβ abrogated the activation of pancreatic stellate cells (PSCs), as observed by reduced collagen-1 (Col-1) release in the TME. Moreover, siRNA adsorbed on the NP surface was found to penetrate tumors and silence PD-L1, which significantly enhanced CD8^+^ T cell infiltration and anti-tumor immune response [283]. These studies rationalized previous clinical trials that focused on concomitant targeting of the PD1-PD-L1 axis and the TGFβ pathway to inhibit ICs and alleviate immunosuppression in PDAC patients (Table 1) [121, 122, 124]. Similarly, inhibition of IL-6 pathway, which is frequently upregulated in PDAC and promotes the TGFβ pathway and its associated immunosuppressive functions, reduced stroma by inhibiting TGFβ1 and Col-1 secretion. In addition, a reduced differentiation of MDSCs was observed after IL-6 blockade, while there was an increase in dendritic cells (DC) maturation, with no difference in M2 macrophage differentiation. This study further showed that targeting IL6 signaling reduced the expression of the PD1-PD-L1 axis on stimulated DCs, suggesting that IL6 inhibition could improve the quality of APCs and synergize with anti-PD1-PD-L1 therapy [284]. Principe et al. showed that the adoptive transfer of TGFβ insensitive CD8^+^ T cells significantly regressed PDAC tumors, which could not be achieved by global inhibition of the TGFβ pathway. Rather, there was an upregulation of PD-L1 after global TGFβ targeting. However, concomitant inhibition of the TGFβ pathway and PD-L1 improved survival and reduced disease-associated morbidity in KPC mice [281]. Similarly, a bifunctional agent, constructed by fusing an anti-PD-L1 antibody with peptide linkers to trap extracellular TGFβ, was found effective in regressing tumors in a preclinical model of PDAC [285]. In the clinical settings, a Phase 1b clinical trial targeting TGFβ and PD-L1 with galunisertib and durvalumab, respectively, showed a tolerable dose-limiting toxicity [286], suggesting that this combination treatment could be used in human PDAC patients (Table 1).

Based on the previously defined immunosuppressive role of regulatory T- and B- cells in PDAC and their association with suppressive cytokines such as IL10, IL18, IL35, IL6, and TGFβ, studies have evaluated combination therapies targeting regulatory T or B cells with anti-PD1 therapy [287–290]. Targeting FoxP3^+^ T-regs and IL35^+^ B-regs in combination with pharmacological inhibition of suppressive cytokines, such as TGFβ and IL6, rendered pancreatic tumors sensitive to anti-PD1 therapy [281, 284, 285]. Other immunosuppressive factors include deregulated chemokines and co-stimulatory molecules that contribute to poor response to ICB agents. Earlier studies suggest that pharmacological inhibition of the CXCR4-CXCL12 axis, which is involved in metastasis, immune infiltration, and poor immunotherapy response [114, 115], improved the outcomes of PD-L1 and PD1-targeted immunotherapies in patients with advanced and metastatic PDAC (Table 1) [137, 291]. On the other hand, the immunosuppressive effects of regulatory T- and B- cells are regulated by the chemokine CCL5. In fact, CCL5 promotes Treg infiltration, and cancer-FoxP3 (C-FoxP3) expressed on these T-regs promotes PD-L1 expression, which rationalizes targeting CCL5 in combination with PD-L1 inhibitors. When administered in a combination regimen, a 3-week treatment with anti-PD-L1 antibody (200 µg/dose/twice a week) enhanced the therapeutic effects of anti-CCL5 antibody significantly [292, 293]. Another study targeted the immunosuppressive CXCL8-CXCR2 axis, predominantly active in CD68^+^ TAMs, in combination with anti-PD1 therapy to determine the effect of the combination immunotherapy on PDAC progression and immunosuppression. Zhang et. al. showed that IFNγ treatment reduced the expression of CXCL8 and enhanced the efficacy of anti-PD1 therapy in a murine model, primarily by mitigating the intra-tumoral infiltration of CD68^+^ M2 macrophages. Intriguingly, anti-PD1 therapy was only effective when administered at an early tumor stage. In contrast, even well-established tumors regressed when ICB therapy was given with IFNγ [112], suggesting that IFNγ may be used as a therapeutic agent in advanced-stage PDAC to improve the efficacy of anti-PD1 therapy.

### Co-stimulatory molecules

The co-stimulatory molecule CD40L is critical for the effective adaptive immunity and is an important target to overcome immunosuppression in PDAC [294–296]. CD40 is expressed on different immune cells and is involved in the activation of both innate and adaptive immune pathways. CD40 agonists such as ligands and antibodies have been well-established for immune activation and generation of polyfunctional T cells in immunologically cold tumors [297, 298]. In addition, CD40 agonists, when combined with inhibitors of immunosuppressive mechanisms regulated by MEK, autophagy, and PD1, improved anti-tumor immune responses and therapeutic efficacy of combination treatments [130, 299]. In fact, treatment with CD40 agonists reduces TAM-mediated immunosuppression and improves the efficacy of immunotherapy [300, 301], particularly the ICB therapies targeting the PD1-PD-L1 axis (Table 1) [130, 131], and therapeutic vaccines [131, 302–304]. Similarly, inhibition of other TAM-associated pathways such as Colony stimulating factor 1/ Colony stimulating factor 1 receptor (CSF1/CSF1R), Phosphatidylinositol 3 kinase (PI3Kγ), and RIPK1 have been demonstrated to reverse immunosuppression and, enhance the therapeutic efficacy of anti-PD1 and anti-CTLA4 immunotherapies in PDAC [305–307]. Targeting the CSF1-CSF1R axis improved the response rate to anti-PD1-PD-L1 therapies, primarily by depleting M2-TAMs and associated immunosuppression (Table 1) [125–127].

Therapeutic strategies targeting metastatic PDAC are limited, different form localized PDAC, and more challenging than targeting primary tumors. Primary tumors often respond better to the treatment than metastatic lesions, primarily due to the difference in their phenotypic, functional, immunological, and metabolic attributes [308, 309]. Although immunotherapy-based treatment modalities have not yet yielded favorable clinical outcomes, there is substantial evidence that emerging PDAC immunotherapies can reverse suppressive immune infiltrates and alleviate immunosuppression at the metastatic *milieu* [309, 310]. Therefore, to make immunotherapy more effective, efforts have been made to mitigate immunosuppression in metastatic PDAC [311–314]. These studies suggest that alleviating immunosuppression in the metastatic microenvironment could improve the efficacy of ICB therapies. For instance, immunosuppressive macrophages release granulin, which is regulated by CSF1 and promotes liver metastasis, T cell exclusion at the metastatic site, and poor response to immunotherapies [48]. Early recruitment of granulin-secreting monocytes activates hepatic stellate cells and promotes fibrotic and immunosuppressive metastatic microenvironment [48]. Moreover, targeting CSF1 reduced granulin release and enhanced anti-PD1 response in the liver metastases model of PDAC [315]. Similarly, a recent study analyzed human and mouse PDAC samples to differentiate the immune landscape and response to PDAC immunotherapies in primary and liver metastases [309]. Several differences in the immune cell composition and response to immunotherapy were observed between primary tumor and liver metastases. First, liver metastases were less responsive to anti-PD-L1 and ICOS agonist therapy compared to primary tumors. Second, liver metastases were enriched in anergic T cells and TAMs (MHCII^lo^IL10^hi^) lacking Ag-presentation machinery compared to primary tumors. Third, regulatory B-cells, recruited by highly abundant MUC1^hi^IL18^hi^ epithelial cells, were most abundant in liver metastases and were found to regulate immunosuppression and response to immunotherapies. Lastly, B-cell depletion or targeting BTLA and CD200 ICs expressed on B cells significantly enhanced the effect of previously failed immunotherapies in targeting liver metastases. This study provided clear evidence that the immunosuppressive *milieu* at the metastatic site is different than in the primary tumor. Therefore, immunotherapies targeting ICs in metastatic PDAC could be more effective if combined with strategies neutralizing immunosuppression.

### Therapeutic vaccines

Therapeutic vaccines have demonstrated appreciable responses in PDAC patients and preclinical models [316–320]. Thus far, whole-cell vaccines, dendritic cell-based vaccines, recombinant proteins, and synthetic peptides have been evaluated in PDAC immunotherapy trials to investigate their efficacy in improving immune response and survival in PDAC [84, 321, 322]. Various PDAC vaccines including a whole cell vaccine (GVAX), MUC1-peptide vaccine, Wilm’s tumor 1 (WT-1) vaccine, algenpantucel-L, mutant KRas peptide vaccine, and *Listeria monocytogenes*-expressing mesothelin (CRS-207) vaccine, have been evaluated in PDAC patients in several trials [84, 317, 322]. Encouraging results have been reported from clinical trials that evaluated allogeneic whole cell GVAX and CRS-207 vaccines in combination with ICB therapies. These studies showed an improved anti-tumor immune infiltrate and proposed this treatment modality suitable as a maintenance therapy for metastatic PDAC patients [323–325]. Recently, mRNA and viral particle-based vaccine formulations have also gained attention for the development of personalized mRNA vaccines [326]. A noteworthy recent study demonstrated that the mRNA-based personalized vaccine having multiple neoepitopes constituted in the same liposomal delivery platform that was used to develop breakthrough SARS-CoV-2 mRNA vaccine is easy to expand and deliver back to the patients in a time-dependent manner [327]. When administered as adjuvant immunotherapy, this mRNA-based personalized vaccine induced strong T cell response specific to neoepitopes, improved the efficacy of chemotherapeutic regimen, and prolonged recurrence-free survival in PDAC patients. However, unlike pathogenic diseases, generating robust and tumor-specific immune response is challenging. Thus far, most clinical trials were performed in metastatic PDAC patients and in combination with standard of care therapy or radiotherapy [84, 317, 322]. Despite a robust response in preclinical models, investigational therapeutic vaccines have failed to generate a clinically significant immune response in patients, warranting a strategy shift before planning future trials. Particularly, therapeutic approaches targeting immunosuppression need to be combined with therapeutic vaccines. Moreover, delivery platforms, such as new-generation nano-polymers that act as co-adjuvants, need to be considered for antigen delivery. Lastly, each patient differs in immunological health and requires personalized vaccines.

## Conclusion and future perspective

Immunosuppression is one of the major factors for PDAC progression, metastasis, and poor immunotherapy response [10, 18]. Neoplasms carrying oncogenic mutations undergo molecular, immunological, and metabolic changes that drive stemness, EMT, MHC-downregulation, and altered secretome, allowing them to gain metastatic traits and survive immune surveillance [16, 56, 238]. The primary tumor site is infiltrated with suppressive immune cells such as M2-TAMs, neutrophils, MDSCs, and regulatory T and B cells that, together with CAFs, contribute to immunosuppressive factors present in the ‘tumor secretome’ [246, 308]. The pancreatic tumor secretome is composed of various cytokines such as TGFβ, IL1β, IL6, IL10, IL17, and IL35 [35, 52, 64, 101, 282, 288], chemokines such as CX3CL1, CXCL12, CCL2, and CCL5, CCL20, CCL21 [50, 110–114, 119, 120, 311], and MMPs such as MMP2, MMP7, and MMP9 [147, 148, 152], which have been recognized as predominant factors contributing to immunosuppression and metastatic PDAC progression. In addition, tumor-secreted vesicles, called exosomes, have been recognized as a key component of the tumor secretome contributing to immunosuppression and PDAC metastasis [42, 161]. In fact, infiltrating cytotoxic T cells, NK cells, and innate immune cells are influenced by these immunosuppressive factors to acquire exhausted or regulatory phenotypes [43, 45, 153, 247, 328, 329]. Thus, suppressive immune cells and the tumor secretome create an immunosuppressive niche at the primary tumor site, promote pro-metastatic features in tumor cells, and support the development of PMNs at metastatic sites.

Circulating tumor cells preferentially metastasize to distant organs depending on the microenvironment in the PMNs, which is considered critical for their organotropic adaptation. As liver is the most common metastatic site in PDAC patients, it has been extensively characterized for molecular and immunological attributes that support PMN development and metastatic growth [45, 330]. In fact, increased expression of STAT3 and serum amyloid proteins and alterations in fibrotic and metabolic pathways promote hepatocyte activation and accumulation of myeloid cells and neutrophils, supporting the development of PMN [49, 267, 331]. However, the type of immune cells that accumulate in, and promote PMNs depends on the target organ for metastasis, as there are reports suggesting that neutrophils are the first cells migrating to and promoting the lung PMNs [43, 45]. In contrast, monocytes predominantly play an early role in liver metastasis [49, 330]. These studies suggest that metastasis-associated macrophages and neutrophils contribute to immunosuppressive PMNs in an organ-specific manner. Together with increased fibrosis, these immune cells provide a conducive microenvironment for CTCs to proliferate within PMNs.

Molecular pathways regulating immunosuppression in PDAC are distinct for each cell type of the TME, which provides an opportunity to develop targeted therapies more precisely. For instance, constitutively activated mutant KRas, Myc, STATs, Wnt/Snail, Notch, and YAP1 are predominantly activated in cancer cells and are known to drive EMT, metastasis, and immunosuppression [23, 26, 66, 72, 86]. In addition, autophagy-induced MHCI downregulation in cancer cells has emerged as an immune escape mechanism, which can be targeted using specific autophagy inhibitors [25]. On the other hand, molecular pathways activated in suppressive immune cells and CAFs are primarily controlled by TGFβ and IL6 in STAT-dependent manner [68, 90, 284]. Particularly, CSF1-CSF1R and CD40-CD40L are key molecular targets regulating the immunosuppressive role of M2-TAMs in metastatic PDAC [297, 300, 306]. Stromal factors such as hypoxia and ECM proteins such as Col-1 and fibronectin are other commonly recognized targets contributing to immunosuppression and metastasis [73, 77, 78, 143, 144]. In addition, kinases such as FAK, RIP1, PKM2, DCLK1, and SRC family kinases such as HCK [218, 224, 227, 228, 257], and molecular pathways associated with nutrient transport and metabolism such as GJB2, AMPK, and mTORC are upregulated in metastatic PDAC and also contribute to immunosuppressive phenotypes [87, 244, 245, 266]. Besides, metabolic checkpoint protein IDO1 expressed on myeloid cells is considered an important target that negatively impacts immunotherapy response in metastatic PDAC [153, 252, 261]. Alterations in the metabolic pathways related to glucose and glutamine synthesis, lipid metabolism, amino acid biosynthesis, and catabolism are also strongly associated with immunosuppression and PDAC metastasis [240, 256]. There is substantial evidence that combined targeting of these molecular and metabolic pathways can be a viable approach to mitigate immunosuppression and enhance the efficacy of current immunotherapies against metastatic PDAC [18, 270, 322].

Recent studies have unraveled the previously unrecognized role of ncRNAs and tumor microbiome in regulating PDAC pathogenesis, immune modulation, and therapy responses [194, 195, 234]. These studies have not only led to greater appreciation of the complex and multilayered mechanisms that drive host-tumor interactions but have also opened new avenues for targeting immunosuppression and metastasis. Based on the differences in the immunosuppressive characteristics of the primary tumor and metastatic microenvironments, combination immunotherapies targeting site-specific immunosuppressive factors can help achieve effective outcomes. Although therapeutic approaches targeting ICs and immunosuppressive factors have shown some promise in PDAC immunotherapy, efforts to mitigate immunosuppression and enhance the response to immunotherapies have been limited. Therefore, approaches to make tumors more immunogenic, develop more potent therapeutic vaccines, select robust antigen delivery platforms and co-adjuvants, and screen patients for personalized immunotherapy, could improve immunotherapy responses. For example, radiation-induced immunogenicity could enhance the efficacy of therapeutic vaccines and ICB therapies by turning immunologically ‘cold’ into ‘hot’ tumors [332–334].

For effective therapeutic vaccines, potent tumor antigens and their delivery platforms are essential for optimal Ag-loading, solubility, persistent Ag-release, and co-adjuvant properties. While modeling mutational changes and characterizing immune alterations have been facilitated by genetically engineered animal models, most animal models fail to recapitulate the neoantigenic profiles of human tumors. Generation of humanized and transgenic mice models or higher vertebrate models expressing human antigens can enable the evaluation of immunotherapies targeting PDAC [335]. Efforts along these lines have led to the development of transgenic MUC1 and CEA mice that have been utilized to evaluate recombinant Ag-vaccine and CAR-T cell-based immunotherapies, respectively [336–338]. In addition, a recently developed glycoengineered mouse model capable of expressing human CA19-9 glycan could be utilized for evaluating CA19-9 targeting antibodies and immunotherapies [339]. Recent advances in nanotechnology provided nano-polymers, nano-liposomes, and virus-based particles, that can serve as robust delivery platforms for therapeutic vaccines [340]. The mRNA vaccine platform used to generate SARS-CoV-2 vaccines to fight the global pandemic gained significant attention for its use in therapeutic cancer vaccines. Recent advances have allowed genomic and transcriptomic profiling of tumors, and bioinformatic tools have enabled the prediction of neoantigens and robust characterization of the immune response. These developments have set the stage for designing personalized vaccines that can be effectively delivered. A recent study showing the efficacy and feasibility of personalized liposomal mRNA vaccine provides future hope for the development of a therapeutic vaccine for PDAC patients [327]. However, evaluating personalized therapeutic vaccines in combination with immunosuppression-targeted therapies will be more insightful in developing combination immunotherapy for PDAC treatment. Besides, using suitable transgenic models or higher vertebrate models could recapitulate PDAC disease and the host immune system, mimicking immunosuppressive hallmarks of aggressive and metastatic human PDAC. Clinical trials in advanced and metastatic PDAC patients have largely failed, primarily due to the high immunosuppression in the patients. Therefore, evaluating immunotherapies in early-stage PDAC patients with an identifiable high risk of developing PDAC, such as patients with pancreatic cystic lesions or individuals with familial PDAC, may be more informative. Moreover, screening methods must be developed to profile likely responders before considering immunotherapy.

## Data Availability

Not applicable, all information in this review can be found in the reference list.

## Abbreviations

3-IAA: 3-Indole-acetic acid
ADAR1: Adenosine deaminase acting on RNA 1
AhR: Aryl hydrocarbon
AMPK: AMP-activated protein kinase
APCs: Antigen presenting cells
Arg1: Arginase 1
ASCT2: Alanine, Serine, Cysteine Transporter 2
BTLA: B and T lymphocyte attenuator
C1QBP: Complement C1q Binding Protein
CA125: Cancer antigen 125
CA19-9: Carbohydrate antigen (*CA) 19-9*
CAFs: Cancer associated fibroblasts
CASC2: Cancer Susceptibility Candidate 2
CCL: CC chemokine ligands
CCR: CC chemokine receptor
CDKN2A: Cyclin Dependent Kinase Inhibitor 2A
CEA: Carcinoembryonic antigen
circRNA: CircularRNA
Col-1: Collagen-1
CSF1-R: Colony-stimulating factor-1 receptor
CTLA4: Cytotoxic T-Lymphocyte Associated Protein 4
CXCRs: C-X-C motif chemokine receptors
DCC: Disseminating cancer cells
DCLK1: Doublecortin Like Kinase 1
DCs: Dendritic cells
ECM: Extracellular matrix
EGFR: Epidermal growth factor receptor
ERK: Extracellular signal-regulated kinases
FAK: Focal adhesion kinase
FZD7: Frizzled Class Receptor 7
GLUT1: Glucose Transporter-1
GPR18: G protein-coupled receptor 18
HCK: Hematopoietic cell kinase
HIF: Hypoxia inducible factor
HSCs: Hepatic stellate cells
HSF1: Heat shock factor 1
ICAM1: Intercellular Adhesion Molecule 1
IFNγ: Interferon-γ
IL: Interleukin
ILCs: Innate lymphoid cells
iNOS: Inducible nitric oxide synthase
JAK2/STAT3: Janus kinase 2/signal transducer and activator of transcription 3
JNK: Jun N-terminal kinase
KCs: Kuffer cells
KLRG1: Killer cell lectin-like receptor G1
KRAS: Kirsten rat sarcoma viral oncogene homolog
Lag3: Lymphocyte activation gene-3
LIF: Leukemia Inhibitory Factor
LIRs: Leukocyte immunoglobulin-like receptors
lncRNA: Long non-coding RNA
MALAT1: Metastasis associated lung adenocarcinoma transcript 1
MDSCs: Myeloid-derived suppressor cells
MEK: Mitogen-activated protein kinase
miR: Micro-RNA
MRC1: Mannose Receptor C-Type 1
MRSA: Methicillin-resistant *Staphylococcus aureus*
mTOR: Mammalian target of rapamycin
MYH9: Myosin Heavy Chain 9
NADPH: Nicotinamide adenine dinucleotide phosphate
ncRNA: Non-coding RNA
NEAA: Non-essential amino acid
NK cell: Natural killer cell
NKG2G: Natural killer group 2 member D protein
Notch 1: Neurogenic locus notch homolog 1
NOX1/2: NADPH oxidase 1/2
PD1: Programmed death-1
PD-L1: Programmed death-ligand 1
PI3K: Phosphoinositide 3-kinase
PIK3: Phosphatidylinositol 3 kinase
PIK3CB: Phosphatidylinositol-4,5-bisphosphate 3-kinase catalytic subunit beta isoform
PKM2: Pyruvate kinase isozyme type M2
PMN: Pre-metastatic niche
RAB11FIP1: Rab11 family-interacting protein *1*
RIP1 kinase: Receptor-interacting serine/threonine-protein kinase-1
SDF1: Stromal cell-derived factor 1
SMAD4: Mothers against decapentaplegic homolog 4
SRC kinase: Src family kinase
STX6: Syntaxin-6
TAM: Tumor-associated macrophage
TCGA: The Cancer Genome Atlas
TDO2: Tryptophan 2,3-dioxygenase
TGF: Transforming growth factor
TIGIT: T cell immunoreceptor with Ig and ITIM domains
TIM3: T-cell immunoglobulin mucin 3
TIMP1: Tissue inhibitors of metalloproteinase-1
TMAO: Trimethylamine N-oxide
TME: Tumor microenvironment
TNFSF9: Tumor Necrosis Factor Receptor Subfamily 9
TP53: Tumor protein 53
VISTA: V-domain Ig suppressor of T cell activation
